# Dynamics of in-hospital body composition changes in mild and moderately severe acute pancreatitis: trajectories and predictors

**DOI:** 10.3389/fnut.2026.1824145

**Published:** 2026-06-04

**Authors:** Dingce Sun, Xin Zhou, Hong Li, Hairong Lin

**Affiliations:** 1 Department of Urology, Mianyang Central Hospital, Mianyang, Sichuan, China; 2 Department of Gastroenterology, Mianyang Central Hospital, Mianyang, Sichuan, China

**Keywords:** body composition, lean tissue mass, muscle wasting, nutrition, pancreatitis

## Abstract

**Objective:**

Nutritional risk is common in pancreatitis, but body composition changes in mild to moderate cases are understudied. This research aimed to track these changes during hospitalization, identify influencing factors, and explore their link to complications like SIRS and peritonitis.

**Methods:**

A prospective observational longitudinal study was conducted, enrolling 206 patients with mild and moderately severe acute pancreatitis admitted to a university-affiliated hospital. Body composition was longitudinally assessed at hospital admission, diet initiation day, and discharge. We employed linear mixed-effects models to analyze the trajectories of body composition changes and their influencing factors in patients with pancreatitis during hospitalization.

**Results:**

During hospitalization, average BMI decreased by 0.9 kg/m^2^, lean tissue mass (LTM) by 1.6 kg, and adipose tissue mass (ATM) by 0.9 kg. Significant changes in both LTM and ATM were observed over time during hospitalization, specifically across admission, diet initiation, and discharge (*p* < 0.05). Multivariable linear mixed-effects model analysis identified length of hospital stay, age, sex, alcohol use history, and hemoglobin level as independent determinants of LTM changes, whereas length of hospital stay, diabetes mellitus, triglyceride level, and ALT were independent determinants for ATM changes. Peritonitis, SIRS, and ascites occurred in 58.5, 12.6, and 4.9% of patients, respectively. In all patients, LTM and ATM showed a consistent decrease with longer hospitalization, but the trends of change did not differ by complication status.

**Conclusion:**

This study reaffirms the necessity of addressing nutritional risk in all pancreatitis patients, as even those with mild and moderate disease experience significant muscle tissue loss.

## 1 Introduction

Acute pancreatitis (AP), a common digestive emergency frequently characterized by abdominal pain and electrolyte imbalances, may progress to multi-organ dysfunction and mortality in severe cases (1). The early disease phase typically manifests a hypermetabolic and hypercatabolic state, leading to negative nitrogen balance and substantial nutritional risk that adversely impacts clinical outcomes (1). The 2020 ESPEN guidelines on clinical nutrition emphasize that all AP patients should be considered at moderate-to-high nutritional risk, with severe AP cases invariably requiring nutritional intervention (2), recommending standardized screening tools such as NRS-2002. An observational study of 1,883 inpatients revealed nutritional risk in approximately 53.3% of pancreatitis cases (3); similarly, 54.3% of severe AP patients demonstrate nutritional risk (4). This risk correlates significantly with alterations in muscle and adipose tissue (5). Critical findings indicate daily skeletal muscle area reductions of 1.1 cm^2^ in severe AP versus 0.5 cm^2^ in moderately severe AP, both associated with prolonged hospitalization (5). Additional studies confirm that a low psoas muscle index correlates with increased extrapancreatic complications, infections, and extended hospital stays (6). In contrast, high visceral adipose tissue combined with low skeletal muscle volume predicts AP severity (7). Nevertheless, while existing research predominantly focuses on severe AP, a paucity of longitudinal evidence characterizes mild-to-moderate presentations (5). Therefore, this study will employ linear mixed-effects models to identify the trajectories of muscle and adipose tissue changes and to determine the influencing factors via fixed and random effects. This approach aims to visualize the nutritional risk in patients with mild-to-moderate disease and identify its determinants, thereby providing a basis for nutritional interventions. Furthermore, we will compare differences in body composition changes among patients with different complications, such as peritonitis, systemic inflammatory response syndrome (SIRS), and ascites.

## 2 Methods

### 2.1 Study design and participants

This single-center, prospective, observational, longitudinal study enrolled patients with acute pancreatitis who were admitted to a university-affiliated hospital in Mianyang, Sichuan, China, between August 2024 and June 2025. Eligible participants were adults diagnosed with mild or moderately severe acute pancreatitis according to the Revised Atlanta classification (1), Mild acute pancreatitis (MAP) is characterized by the absence of organ failure as well as local or systemic complications. Moderately severe acute pancreatitis (MSAP) is defined by the presence of transient organ failure lasting less than 48 h or local or systemic complications, but without persistent organ failure. All included patients had clear consciousness and no communication barriers. Exclusion criteria comprised unwillingness to participate and a pancreatitis course exceeding 24 h.

### 2.2 Data collection

Demographic (age, sex, body mass index [BMI]), clinical history (AP type, comorbidities, severity), and laboratory parameters were collected at admission, including Inflammatory markers: Procalcitonin (PCT), C-reactive protein (CRP), white blood cell (WBC), Red Blood Cell (RBC), Hemoglobin, Neutrophils, Liver function tests: Alanine aminotransferase (ALT), Aspartate transaminase (AST); Lipid profiles: Total cholesterol (TC), triglyceride (TG), and high-density lipoprotein cholesterol (HDL-C), low-density lipoprotein cholesterol (LDL-C); Pancreatic enzymes: Serum amylase, lipase.

Body composition was measured using a bioimpedance device (BCM Body Composition Monitor, Fresenius Medical Care, Shanghai). Parameters included BMI, lean tissue mass (LTM), and adipose tissue mass (ATM). Standardized protocol required participants to: remove metal objects/heavy clothing, rest supine for 5–8 min, with electrodes applied to the ipsilateral hand/foot dorsa.

### 2.3 Nutrition regimen

Initially, upon admission, the patient received daily parenteral nutrition consisting of 500 mL of amino acids (containing 42 g) plus 1,500 mL of 5% GNS (containing 75 g of glucose), providing approximately 468 kcal/day. After the patient’s symptoms, particularly abdominal pain, were markedly relieved or resolved (with a VAS score below 3), and no nausea or vomiting occurred, oral intake was initiated. At that point, the nutritional regimen was adjusted to a combination of oral feeding and parenteral nutrition, without any other form of enteral nutrition (i.e., no tube feeding). The revised regimen included daily intravenous infusion of 500 mL of amino acids (42 g) plus 1,000 mL of 5% GNS (50 g glucose), along with 300 g of plain rice porridge (240 kcal) and 200 g of steamed bread (350 kcal), resulting in a total calculated energy intake of approximately 960 kcal/day.

### 2.4 Data collection timeline

Baseline data were collected at admission. Body composition assessments were performed at three timepoints: admission (T1), diet initiation day (T2), and discharge (T3). T2 was defined as the first calendar day on which the patient resumed oral intake (clear liquid diet) after a period of exclusive parenteral nutrition, based on the following predefined criteria: abdominal pain visual analog scale (VAS) ≤ 3, absence of nausea or vomiting, presence of bowel sounds, and the patient’s expressed willingness to eat. All patients received exclusive parenteral nutrition support prior to T2.

### 2.5 Sample size

The sample size was calculated using the formula for one-way repeated measures ANOVA. Here, *M* denotes the required sample size, and *K* represents the number of repeated measurements. Parameters were set as follows: *K* = 3, *α* = 0.05, *β* = 0.20 (two-tailed test), *Z*₁-α/₂ = 1.96, *Z*₁-_β_ = 0.842, and the correlation coefficient between successive measurements *ρ* = 0.7. The standard deviation (*σ*) was 8.1 kg, based on the mean LTM value of 35.5 kg at T3. The minimum detectable difference (*δ*) was set at 10%. Accounting for a 10% attrition rate, the final required sample size was 37. This study included 206 participants, exceeding the calculated requirement.


$$M=[1+(K-1)\rho ]\frac{\sigma^{2}{\left(Z_{1-\alpha /2}+Z_{1-\beta }\right)}^{2}}{k\delta^{2}}$$

### 2.6 Statistical analysis

For the limited missing data (<5%), mean or median imputation was applied. Statistical analyses were conducted using SPSS 19.0 and R 4.1.2, with categorical variables as *n* (%). The study employed linear mixed-effects models to analyze longitudinal changes in LTM and ATM. Each model included a patient-specific random intercept to account for within-individual correlations. Fixed effects comprised time points, along with sociodemographic and laboratory test variables. To adjust for the influence of varying measurement intervals, all models incorporated the period from T1 to T2 (fasting duration) and from T2 to T3 (refeeding duration) as covariates. In subgroup comparisons, a time-by-group interaction term was added to test for differences in the trajectories of change between groups. For univariate screening, a threshold of *p* < 0.05 was used for entry into the multivariate model.

## 3 Results

### 3.1 Basic patient information

A total of 206 patients were included in this study, of which 62.6% were male, and other data are shown in Table 1.

**Table 1:** Basic information table of patients with pancreatitis (*N* = 206).

Item	*N*(%)	LTM		ATM	
		*t*	*p*	*t*	*p*
Age (year)		−4.730	<0.01*	−1.099	0.27
≥60	34(16.5%)				
<60	172(83.5%)				
Gender		13.069	<0.01*	1.411	0.16
Male	129(62.6%)				
Female	77(37.4%)				
Type		3.492	<0.01*	2.849	<0.01*
Biliary	26(12.6%)				
Hyperlipidemic	90(43.7%)				
Alcoholic	20(9.7%)				
Idiopathic	70(34.0%)				
Number of episodes		1.745	0.08	−2.037	0.04*
Initial onset	121(58.7%)				
Recurrence	85(41.3%)				
Smoking history		5.607	<0.01*	1.598	0.11
No	121(58.7%)				
Yes	85(41.3%)				
Alcohol history		3.776	<0.01*	1.707	0.09
No	107(51.9%)				
Yes	99(48.1%)				
Severity of AP		−1.761	0.08	0.982	0.33
Mild	154(74.8%)				
Moderate	52(25.2%)				
Diabetes		−0.284	0.78	2.471	0.01*
No	168(81.6%)				
Yes	38(18.4%)				
COPD		−1.574	0.12	−1.323	0.19
No	202(98.1%)				
Yes	4(1.9%)				
Liver disease		0.745	0.46	−0.704	0.48
No	187(90.8%)				
Yes	19(9.2%)				
WBC (10^9/L)		1.874	0.06	2.397	0.02*
<3.5	1(0.5%)				
3.5–9.5	53(25.7%)				
>9.5	152(73.8%)				
Neutrophil (10^9/L)		2.171	0.03*	0.929	0.35
<1.8	2(1.0%)				
1.8–6.3	37(18.0%)				
>6.3	167(81.1%)				
Hb (g/L)		−2.520	0.01*	4.434	<0.01*
Low	42(20.4%)				
Normal	151(73.3%)				
High	13(6.3%)				
Albumin (g/L)		1.698	0.09	1.535	0.13
<40	34(16.5%)				
40–55	170(82.5%)				
>55	2(1.0%)				
HDL-C (mmol/L)		−5.172	<0.01*	−2.693	0.01*
≤1.04	129(62.6%)				
>1.04	77(37.4%)				
LDL-C (mmol/L)		−1.975	0.04*	−0.464	0.64
<3.4	189(91.7%)				
≥3.4	17(8.3%)				
PCT (μg/L)		−0.371	0.71	2.395	0.02*
0–0.05	40(19.4%)				
>0.05	166(80.6%)				
CRP (mg/L)		1.194	0.23	0.765	0.45
0–6	75(36.4%)				
>6	131(63.6%)				
RBC (10^12/L)		1.448	0.15	3.008	<0.01*
<4.3	49(23.8%)				
4.3–5.8	152(73.8%)				
>5.8	5(2.4%)				
ALT (U/L)		−0.922	0.36	2.629	0.01*
<7	9(4.4%)				
7–40	147(71.4%)				
>40	50(24.3%)				
AST (U/L)		−1.377	0.17	1.084	0.28
<13	21(10.2%)				
13–35	141(68.4%)				
>35	44(21.4%)				
TC (mmol/L)		3.322	<0.01*	2.242	0.03*
<5.18	112(54.4%)				
≥5.18	94(45.6%)				
TG (mmol/L)		4.837	<0.01*	4.164	<0.01*
<1.7	64(31.1%)				
≥1.7	142(68.9%)				
Cr (μmol/L)		−1.555	0.12	1.969	0.05
<57	52(25.2%)				
57–111	149(72.3%)				
>111	5(2.4%)				
Amylase (U/L)		−1.510	0.13	−0.478	0.63
<35	8(3.9%)				
35–135	65(31.6%)				
>135	133(64.6%)				
Lipase (U/L)		1.295	0.20	0.408	0.68
13–60	24(11.7%)				
>60	182(88.3%)				

BMI, body mass index; LTM, lean tissue mass; ATM, adipose tissue mass; AP, acute pancreatitis; PCT, procalcitonin; CRP, C-reactive protein; ALT, alanine aminotransferase; AST, aspartate transaminase; TC, total cholesterol; TG, triglycerides; Cr, creatinine; WBC, white blood cell; HDL-C, high-density lipoprotein cholesterol; LDL-C, low-density lipoprotein cholesterol; Hb, hemoglobin; RBC, red blood cell. *P < 0.05.

### 3.2 Trajectory of body composition changes in pancreatitis patients

**Figure 1:**
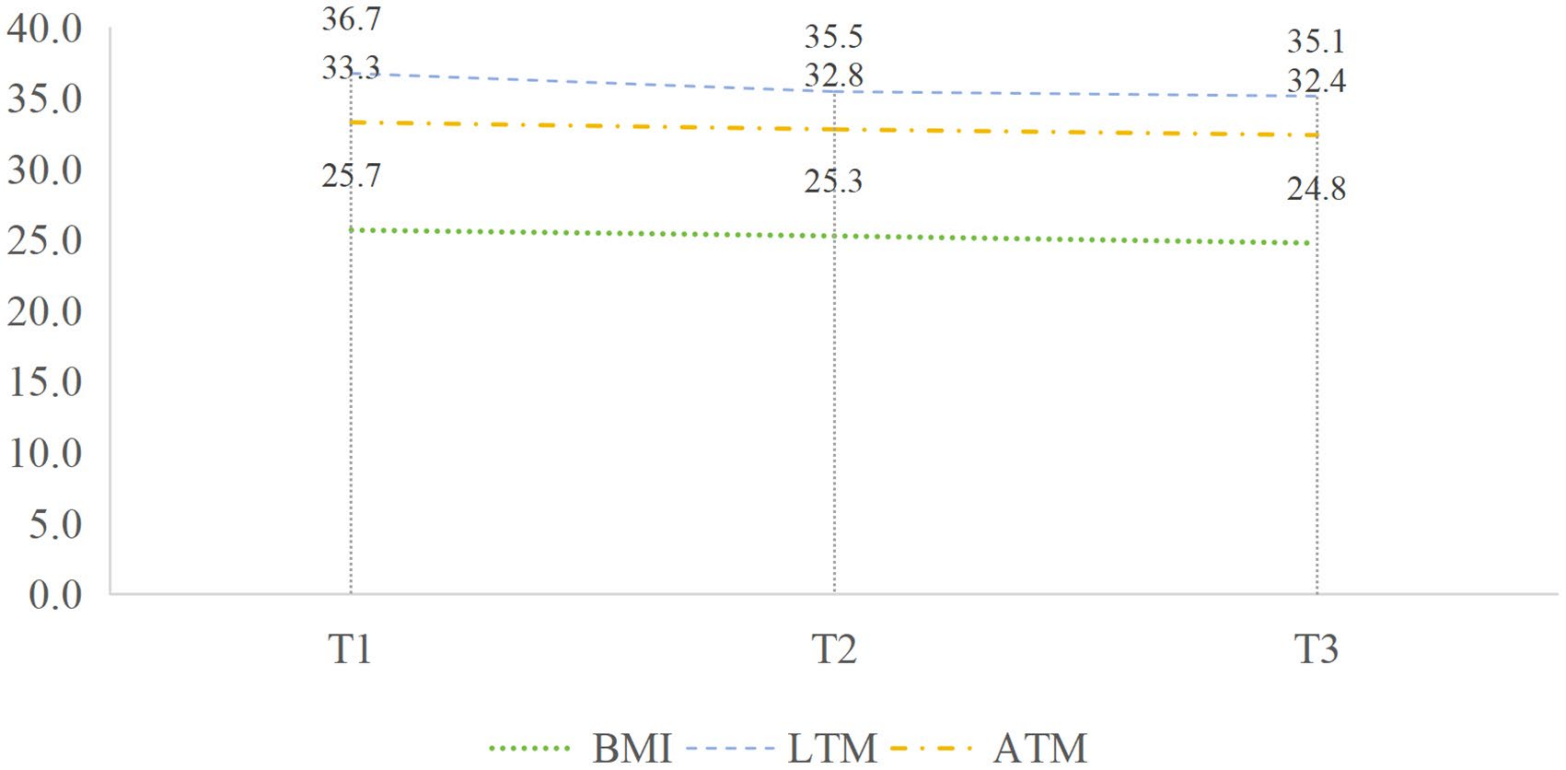
The trajectory of body composition of patients with pancreatitis during hospitalization. T1: On admission; T2: Open eating day; T3: At discharge; BMI: Body mass index (kg/m^2^); LTM: Lean tissue mass (kg); ATM: Adipose tissue mass (kg).

The cohort presented with an initial body mass index (BMI) of 25.7 kg/m^2^, LTM of 36.7 kg, and ATM of 33.3 kg upon hospital admission. The study demonstrated a consistent declining trajectory in BMI, LTM, and ATM throughout the hospitalization period. This decline persisted, albeit at a diminished rate, following the commencement of oral intake. On average, reductions amounted to 0.9 kg/m^2^ in BMI, 1.6 kg in LTM, and 0.9 kg in ATM. Notably, LTM exhibited the most pronounced alteration, while ATM remained relatively stable (Figure 1).

**Figure 2:**
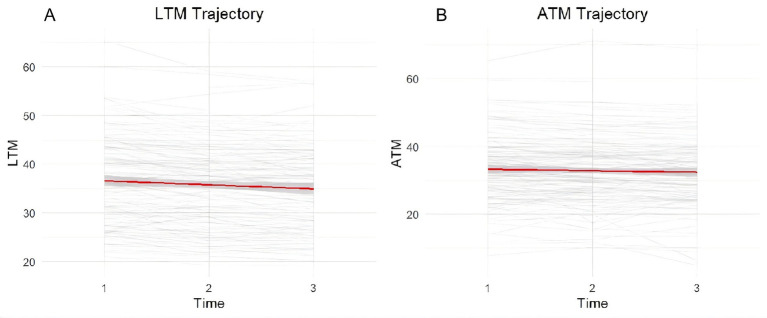
Changes in body composition during hospitalization in patients with acute pancreatitis. **(A)** Changes in lean tissue mass (LTM, kg) over time. **(B)** Changes in adipose tissue mass (ATM, kg) over time. Time points 1, 2, and 3 represent admission, oral refeeding, and discharge, respectively.

Patients with pancreatitis demonstrated a declining trend in the mean values of both LTM and ATM, with the change in LTM being more pronounced than that in ATM (see Figure 2).

### 3.3 Trend analysis of body composition changes in patients with different complications

**Figure 3:**
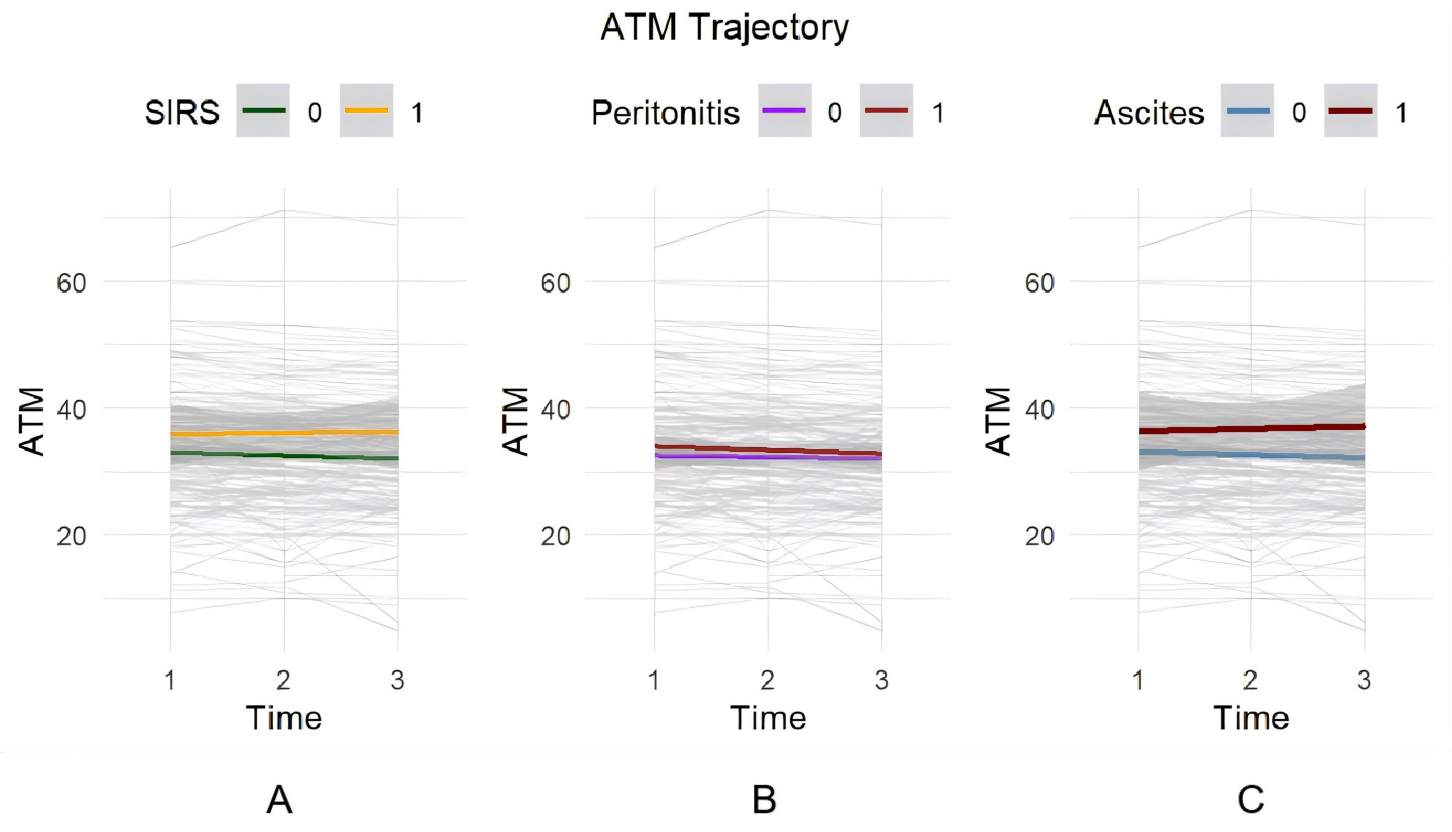
Changes in LTM during hospitalization, stratified by complications. **(A)** LTM changes in patients with or without systemic inflammatory response syndrome (SIRS). **(B)** LTM changes in patients with or without peritonitis. **(C)** LTM changes in patients with or without ascites. Time points 1, 2, and 3 indicate admission, oral refeeding, and discharge, respectively. Values of 0 and 1 represent the absence or presence of the complication, respectively.

**Figure 4:**
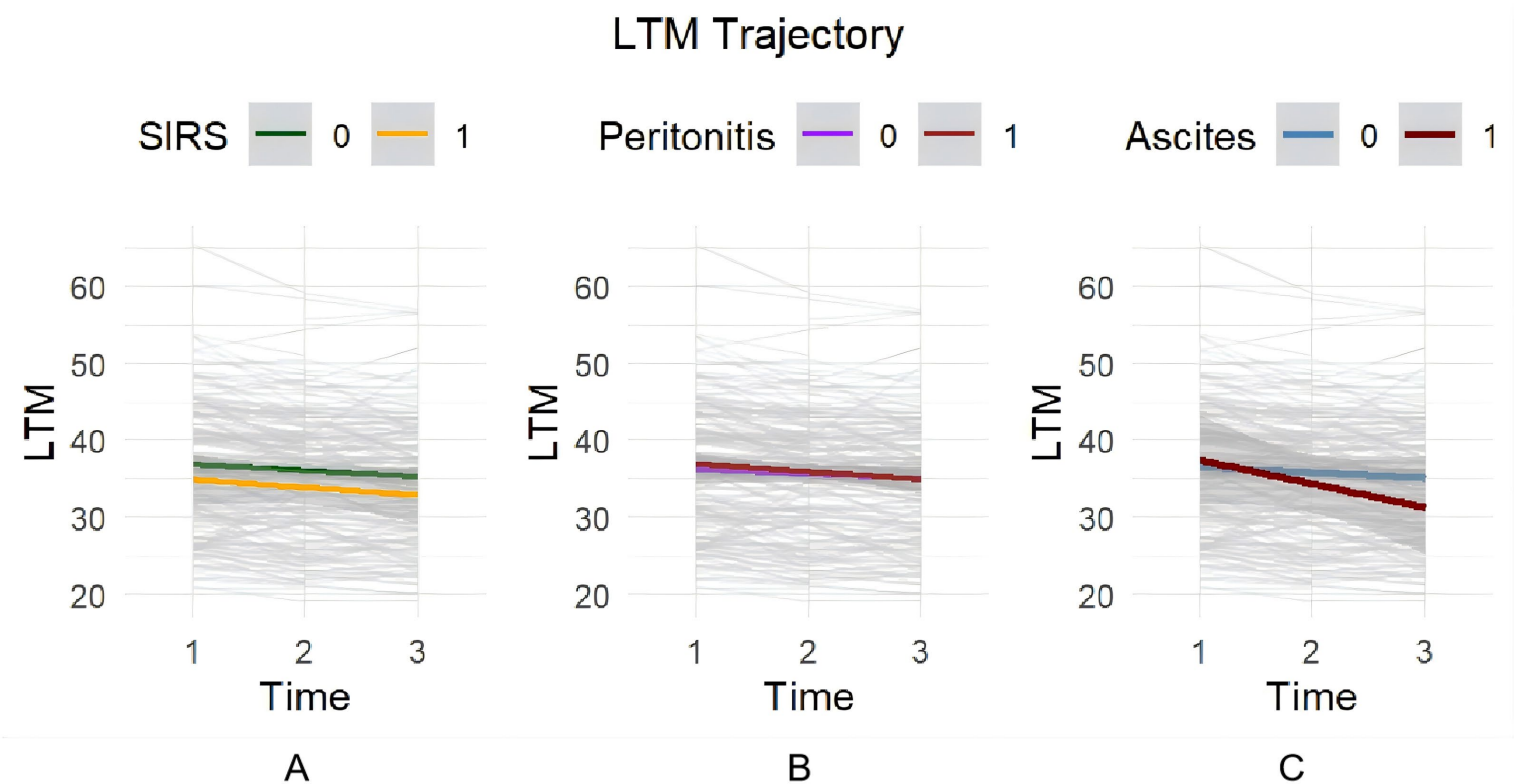
Changes in ATM during hospitalization, stratified by complications. **(A)** ATM changes in patients with or without SIRS. **(B)** ATM changes in patients with or without peritonitis. **(C)** ATM changes in patients with or without ascites. Time points 1, 2, and 3 indicate admission, oral refeeding, and discharge, respectively. Values of 0 and 1 represent the absence or presence of the complication, respectively.

The study identified that 121 (58.5%) patients with pancreatitis developed peritonitis, 26 (12.6%) developed systemic inflammatory response syndrome (SIRS), and 10 (4.9%) developed ascites. Regardless of the presence of SIRS, peritonitis, or ascites, the lean tissue mass (LTM) and abdominal tissue mass (ATM) of acute pancreatitis (AP) patients consistently decreased as the length of hospitalization increased (*p* < 0.05). However, there was no significant difference in the trends of LTM and ATM changes between patients with or without complications (*p* > 0.05) (Figures 3, 4).

### 3.4 Analysis of factors influencing differential trajectories of body composition change in pancreatitis patients

#### 3.4.1 Factors from univariable analysis

To identify factors associated with body composition, univariable linear mixed-effects models were fitted. The results, detailed in Table 1, showed that significant factors (*p* < 0.05) for LTM included age, sex, pancreatitis type, smoking and alcohol history, neutrophil count, hemoglobin, and lipid profiles (HDL-C, LDL-C, total cholesterol, triglycerides). For ATM, significant factors were pancreatitis type, episode frequency, diabetes, white blood cell count, hemoglobin, HDL-C, PCT, red blood cell count, ALT, total cholesterol, and triglycerides (Table 2).

**Table 2:** The differences in LTM and ATM changes in acute pancreatitis patients with or without comorbidities.

Item	ATM				LTM			
	Estimate	SE	*t*	*p*	Estimate	SE	*t*	*p*
(Intercept)	33.51	1.84	18.21	<0.001*	41.71	1.59	26.26	<0.001*
Time 2	−0.53	0.31	−1.70	0.090	−1.13	0.24	−4.78	<0.001*
Time 3	−0.99	0.32	−3.10	0.002*	−1.92	0.24	−7.99	<0.001*
SIRS	3.26	2.14	1.52	0.129	−1.48	1.86	−0.80	0.427
Time 2: SIRS	−1.13	0.90	−1.26	0.209	−0.21	0.69	−0.30	0.762
Time 3: SIRS	−0.94	0.94	−1.00	0.319	1.15	0.72	1.60	0.111
(Intercept)	32.76	1.96	16.73	<0.001*	41.12	1.69	24.27	<0.001*
Time 2	−0.31	0.45	−0.68	0.497	−0.94	0.35	−2.69	0.008*
Time 3	−0.73	0.45	−1.60	0.110	−1.61	0.34	−4.71	<0.001*
Peritonitis	1.61	1.44	1.12	0.262	1.17	1.24	0.95	0.345
Time 2: Peritonitis	−0.61	0.59	−1.04	0.301	−0.37	0.45	−0.82	0.413
Time 3: Peritonitis	−0.66	0.61	−1.10	0.274	−0.33	0.46	−0.72	0.471
(Intercept)	33.93	1.89	17.98	<0.001*	41.95	1.62	25.86	<0.001*
Time 2	−0.70	0.30	−2.34	0.019*	−1.13	0.23	−4.93	<0.001*
Time 3	−1.07	0.31	−3.48	<0.001*	−1.79	0.23	−7.66	<0.001*
Ascites	3.09	3.43	0.90	0.369	2.59	2.97	0.87	0.386
Time 2: Ascites	0.73	1.32	0.56	0.580	−0.59	1.05	−0.56	0.577
Time 3: Ascites	−0.61	1.38	−0.44	0.662	−0.18	1.10	−0.17	0.868

*P < 0.05. This model has adjusted T1 to T2 (fasting duration) and from T2 to T3 (refeeding duration).

#### 3.4.2 Independent determinants from multivariable analysis

A subsequent multivariable analysis, incorporating all significant univariable factors, was performed to isolate independent predictors. The multivariable model demonstrated that hospital stay duration, age, sex, alcohol history, and hemoglobin were independent determinants for LTM (Table 3). Independently, hospital stay duration, diabetes, triglycerides, and ALT were significant for ATM (Table 4).

**Table 3:** Factors associated with LTM change trajectories.

Term	Estimate	Std_Error	*t*_value	*p*_value	95%CI
(Intercept)	31.12	2.40	12.97	<0.001*	(26.38, 35.85)
Time 2	−1.16	0.22	−5.23	<0.001*	(−1.60, −0.73)
Time 3	−1.79	0.23	−7.89	<0.001*	(−2.24, −1.35)
Male	11.65	1.12	10.40	<0.001*	(9.44, 13.86)
Smoking history	0.25	1.05	0.24	0.813	(−1.83, 2.33)
HDL-C (Low)	−0.74	0.80	−0.93	0.353	(−2.31, 0.83)
TG (Low)	0.48	0.94	0.51	0.608	(−1.37, 2.34)
Age ≥ 60 (years)	−4.75	1.19	−4.00	<0.001*	(−7.09, −2.40)
Alcohol history	−1.95	0.99	−1.96	0.05*	(−3.91, 0.01)
Hyperlipidemic AP	−1.02	1.92	−0.53	0.597	(−4.82, 2.78)
Idiopathic AP	−2.55	1.48	−1.72	0.087	(−5.48, 0.38)
Alcoholic AP	−2.61	1.92	−1.36	0.176	(−6.39, 1.18)
TC (Low)	1.02	0.88	1.16	0.249	(−0.72, 2.77)
Hb (Low)	−2.67	1.44	−1.85	0.066	(−5.52, 0.17)
Hb (Normal)	−1.91	0.89	−2.14	0.034*	(−3.66, −0.15)
Neutrophil (Low)	4.44	3.10	1.43	0.153	(−1.66, 10.55)
Neutrophil (Normal)	−3.38	1.92	−1.76	0.080	(−7.16, 0.41)
LDL-C (LOW)	−1.02	1.15	−0.88	0.378	(−3.29, 1.25)

LTM, lean tissue mass; AP, acute pancreatitis; TC, total cholesterol; TG, triglycerides; HDL-C, high-density lipoprotein cholesterol; Hb, hemoglobin. This model has adjusted T1 to T2 (fasting duration) and from T2 to T3 (refeeding duration). *P < 0.05.

**Table 4:** Factors associated with ATM change trajectories.

Term	Estimate	Std_error	*t*_value	*p*_value	95%CI
(Intercept)	31.60	4.87	6.49	<0.001*	(21.99, 41.20)
Time 2	−0.66	0.29	−2.26	0.025*	(−1.23, −0.08)
Time 3	−1.10	0.30	−3.66	<0.001*	(−1.69, −0.51)
Hb (Low)	2.92	2.92	1.00	0.319	(−2.84, 8.68)
Hb (Normal)	1.01	1.66	0.61	0.543	(−2.26, 4.29)
TG (Low)	2.92	1.44	2.03	0.044*	(0.08, 5.76)
RBC (Low)	4.87	3.74	1.30	0.195	(−2.51, 12.25)
RBC (Normal)	0.68	2.19	0.31	0.758	(−3.65, 5.00)
Hyperlipidemic AP	4.53	2.98	1.52	0.130	(−1.35, 10.42)
Idiopathic AP	3.24	2.39	1.35	0.177	(−1.48, 7.96)
Alcoholic AP	−0.74	2.91	−0.25	0.800	(−6.47, 4.99)
HDL-C (LOW)	−0.12	1.19	−0.10	0.918	(−2.48, 2.23)
ALT (Low)	7.06	2.55	2.77	0.006*	(2.04, 12.08)
ALT (Normal)	−1.97	1.53	−1.28	0.201	(−4.99, 1.06)
Diabetes	3.67	1.73	2.12	0.035*	(0.26, 7.09)
WBC (Low)	−7.51	7.00	−1.07	0.285	(−21.31, 6.29)
WBC (Normal)	4.29	4.19	1.03	0.306	(−3.96, 12.55)
PCT (Low)	1.69	1.17	1.45	0.149	(−0.61, 4.00)
TC (Low)	−0.64	1.31	−0.49	0.628	(−3.22, 1.95)
Recurrence	−1.91	1.38	−1.38	0.170	(−4.63, 0.82)

This model has adjusted T1 to T2 (fasting duration) and from T2 to T3 (refeeding duration). BMI, body mass index; LTM, lean tissue mass; ATM, adipose tissue mass; AP, acute pancreatitis; PCT, procalcitonin; CRP, C-reactive protein; ALT, alanine aminotransferase; AST, aspartate transaminase; TC, total cholesterol; TG, triglycerides; Cr, creatinine; WBC, white blood cell; HDL-C, high-density lipoprotein cholesterol; LDL-C, low-density lipoprotein cholesterol; Hb, hemoglobin; RBC, red blood cell. *P < 0.05.

## 4 Discussion

### 4.1 Analysis of in-hospital body composition trajectories in pancreatitis patients

This study documented a consistent decline in BMI, LTM, and ATM among pancreatitis patients during hospitalization. This negative trajectory continued even after the initiation of oral feeding, though the rate of decline attenuated compared to the exclusive parenteral phase. Mean reductions were 0.9 kg/m^2^ for BMI, 1.6 kg for LTM (4.3% loss), and 0.9 kg for ATM.

Data on body composition changes—particularly muscle mass—in mild to moderately severe acute pancreatitis are scarce. In severe ICU cases, substantial muscle loss has been reported: one study found a daily reduction of 0.5 cm^2^ in muscle cross-sectional area during the first 3 weeks of ICU stay (5); another reported a decrease in psoas muscle area from 17.08 cm^2^ to 9.61 cm^2^ at discharge (40.2% reduction) (8). The smaller LTM loss in our cohort likely reflects milder disease severity and the use of whole-body bioelectrical impedance analysis versus localized CT measurements. For context, a comparable LTM loss (2 kg) was observed in non-small cell lung cancer patients over 3 months of chemotherapy (9), and a 5% short-term muscle loss meets the diagnostic threshold for protein-energy wasting in nephrology (10). These comparisons suggest that even patients with mild-to-moderate acute pancreatitis may experience clinically relevant muscle loss.

The observed muscle loss arises from two intertwined mechanisms, both rooted in the disease process. First, disease-induced hypercatabolism: pancreatitis triggers a stress state that elevates glucose demand. Given limited glycogen stores and reduced intake due to early fasting, a mismatch occurs. The body compensates via gluconeogenesis, using amino acids from muscle protein breakdown as substrates, leading to rapid muscle loss and negative nitrogen balance (1). Muscle mobilization is more efficient than fat mobilization, so muscle depletion exceeds fat loss. Inflammatory cytokines further promote protein degradation and suppress synthesis, accelerating the decline in muscle mass and function (11).

Second, disease-mandated nutritional restriction due to feeding intolerance and contraindication to intravenous lipids. Upon admission, nil-by-mouth patients received parenteral nutrition, providing 468 kcal/day. Intravenous lipid emulsions were withheld because hypertriglyceridemia contraindicates their use in hyperlipidemic pancreatitis. After symptom resolution, oral intake began at a mean of 3.33 days. The regimen was then adjusted to combined oral feeding and continued parenteral nutrition, yielding ~960 kcal/day. The mean time to oral diet initiation in this cohort contrasts with current guideline recommendations advocating for early oral feeding within 24–72 h if tolerated (2, 12). Early enteral nutrition is emphasized for its role in preserving intestinal mucosal barrier integrity, mitigating bacterial translocation, and consequently reducing the risk of infectious pancreatic necrosis, organ failure (13), and potentially shortening hospital length of stay and lowering costs (14). However, adherence to these feeding guidelines remains suboptimal, with studies indicating compliance rates as low as 41.9%, particularly in Asian and American settings, underscoring the need for shifts in clinical practice paradigms (15). Inadequate energy provision from parenteral nutrition is largely due to feeding intolerance, which occurs in up to 25% of acute pancreatitis patients and is associated with more severe disease course and poorer clinical outcome (16). Furthermore, current protocols for transitioning to oral intake in pancreatitis often commence with low-fat, soft diets (17) but lack clarity regarding optimal caloric and nutrient targets and structured refeeding schedules, highlighting an area requiring further investigation.

This study innovatively demonstrates that clinically significant muscle loss occurs even in mild-to-moderately severe acute pancreatitis, reinforcing guideline assertions that nutritional risk is present across all pancreatitis types, including those predicted to be mild (17). Supporting the long-term impact, a follow-up study of severe acute pancreatitis patients found that after a mean period of 438.73 days, while 81% showed some increase in muscle mass, only 27% achieved full recovery compared to pre-morbid status; the majority still exhibited a substantial deficit (mean: −31.96%) (8). These findings collectively emphasize the critical importance of nutritional risk screening and timely intervention for all pancreatitis patients, irrespective of initial severity assessment.

### 4.2 Analysis of factors affecting LTM

Our findings indicate that female sex is a risk factor for muscle loss, potentially due to fundamental differences in body composition. For a given BMI, females generally possess a lower proportion of muscle and a higher proportion of fat compared to males (18). The underlying mechanisms involve sex-specific hormonal regulation: estrogen enhances insulin sensitivity and promotes subcutaneous fat deposition (19), whereas testosterone can induce insulin resistance and facilitate visceral fat accumulation (20).

Our results indicate that younger age is protective against muscle loss, while older age is a significant risk factor. Sarcopenia is prevalent in 10–16% of the global elderly population, with rising incidence (21). Age-related mitochondrial dysfunction—characterized by DNA damage, elevated ROS, and impaired biogenesis and quality control—is a key driver, disrupting energy metabolism and compromising muscle integrity (22). As shown by Sataranatarajan et al. (23), aging promotes mitochondrial stress and ROS overproduction, causing oxidative damage to proteins. When combined with the age-related decline in synthetic capacity and frequent nutritional deficiencies, these factors collectively drive the inevitable decline in muscle mass and strength observed in the elderly (23).

A history of alcohol use was identified as a risk factor for muscle loss. Approximately 40–60% of high-risk drinkers exhibit skeletal muscle maladaptation (24). This may be attributed to the acute effects of alcohol, which can impair and dysregulate the signaling of the mechanistic target of rapamycin complex 1 (mTORC1), leading to insufficient muscle protein synthesis and subsequent atrophy (25). Furthermore, chronic ethanol exposure adversely affects mitochondrial function in skeletal muscle, thereby hindering muscle anabolism (26). Other potential mechanisms include impaired satellite cell function, disruption of circadian rhythms, and epigenetic adaptations (24).

Lower hemoglobin levels were significantly associated with muscle loss in our study. This is consistent with evidence linking low Hb to poor muscle metrics in the elderly (27). The mechanism is likely twofold: (1) Chronic hypoxia from anemia directly compromises skeletal muscle function (28), and (2) Low-grade inflammation associated with anemia induces insulin resistance (27). This resistance blunts insulin’s suppression of protein breakdown (proteolysis), disrupting the synthesis-breakdown equilibrium and culminating in muscle atrophy (29).

### 4.3 Analysis of factors affecting ATM

Patients with diabetes experience more significant changes in ATM. In this study, all patients had type 2 diabetes, a condition commonly associated with abnormal glucose metabolism, which leads to increased fat breakdown. Gillies et al. (30) also found that after an acute pancreatitis attack, patients with glucose metabolism abnormalities had significantly elevated serum triglycerides and glycerol, which may be related to fat breakdown. Patients with low triglyceride levels show more drastic changes in ATM. Acute pancreatitis is characterized by a high metabolic state, requiring more energy, leading to increased fatty acid breakdown. However, with lower triglyceride levels, the synthesis is insufficient, resulting in greater ATM loss. ALT is typically a marker of liver damage. The relationship between ALT levels and changes in adipose tissue in patients with acute pancreatitis has been rarely investigated and therefore deserves further exploration.

### 4.4 Strengths and limitations

This study innovatively quantifies nutritional risk in mild and moderately severe acute pancreatitis, employing BIA to demonstrate significant short-term muscle depletion, necessitating nutritional risk screening and early intervention. Limitations include its single-center design and modest sample size, potentially limiting generalizability; multicenter studies are warranted to enhance external scalability. Furthermore, while we minimized the influence of fluid status through standardized measurements and outlier exclusion, BIA remains indirectly affected by hydration. Future studies using multi-frequency BIA devices that can estimate extracellular water and correct for fluid overload, or employing CT, would provide more accurate body composition trajectories in acute pancreatitis.

## 5 Conclusion

Patients with mild to moderate acute pancreatitis experience significant muscle loss during hospitalization, reflecting a high nutritional risk. This indicates the need for early nutritional risk screening and management for these patients. Factors influencing LTM include age, gender, alcohol history, and hemoglobin levels, while factors affecting ATM changes include diabetes, triglycerides, and ALT levels. These risk factors should be considered when designing targeted nutritional interventions.

## Data Availability

The raw data supporting the conclusions of this article will be made available by the authors, without undue reservation.
