# Supplementary figures and images for: Dynamics of in-hospital body composition changes in mild and moderately severe acute pancreatitis: trajectories and predictors
Source: Front Nutr. 2026 Jun 4;13:1824145. doi: 10.3389/fnut.2026.1824145 (PMC13275276; doi:10.3389/fnut.2026.1824145)

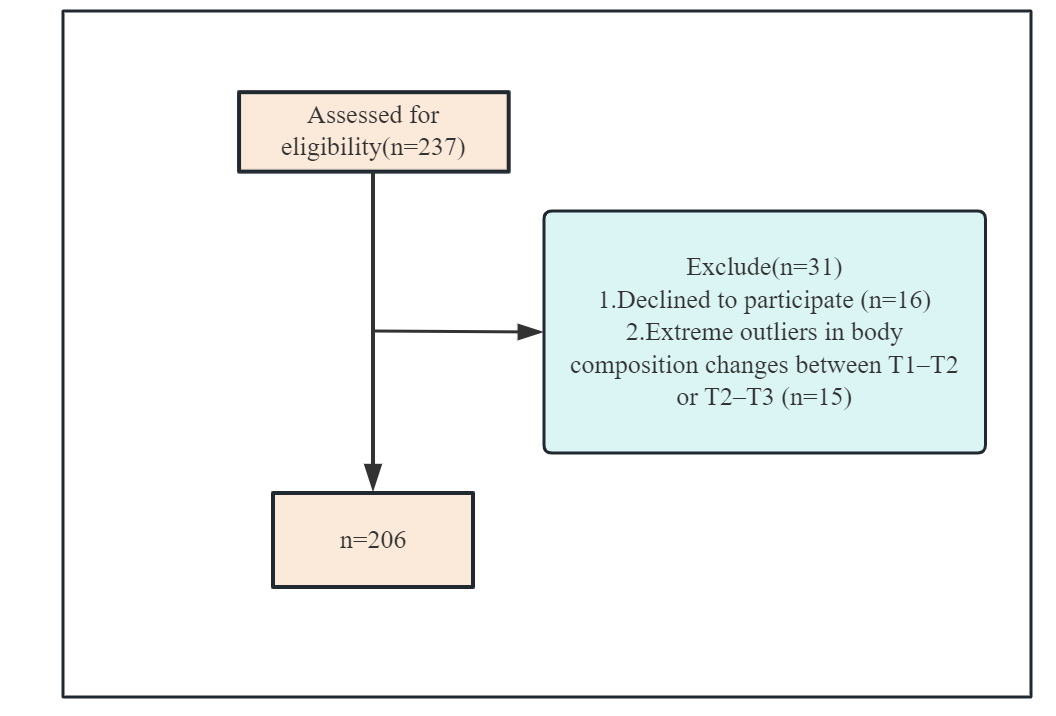

Supplement: Supplementary file 1 [file Image_1.png]
